# Urine lipoarabinomannan for rapid tuberculosis diagnosis in HIV-infected adult outpatients in Khayelitsha

**DOI:** 10.4102/sajhivmed.v22i1.1226

**Published:** 2021-04-26

**Authors:** Bianca Sossen, Amanda Ryan, Joanna Bielawski, Riana Greyling, Gillian Matthews, Sheetal Hurribunce-James, René Goliath, Judy Caldwell, Graeme Meintjes

**Affiliations:** 1 Department of Medicine, Faculty of Health Sciences, University of Cape Town, Cape Town, South Africa; 2 Wellcome Centre for Infectious Diseases Research in Africa, Institute of Infectious Disease and Molecular Medicine, University of Cape Town, Cape Town, South Africa; 3 Town 2 Clinic, Cape Town City Health Department, Cape Town, South Africa; 4 Cape Town City Health Department, Cape Town, South Africa; 5 Matthew Goniwe Clinic, Cape Town City Health Department, Cape Town, South Africa

**Keywords:** tuberculosis, lipoarabinomannan, ambulatory, outpatient, point-of-care, urine, HIV, diagnostic

## Abstract

**Background:**

Decreasing tuberculosis (TB) mortality is constrained by diagnostic and treatment delays. The World Health Organization (WHO) recently actively recommended the point-of-care Alere Determine Lipoarabinomannan Ag assay (AlereLAM) to assist in the diagnosis of tuberculosis in specific HIV-infected outpatients.

**Objectives:**

The primary objective of this study was to compare time to ambulatory TB treatment in HIV-infected adults with CD4 ≤ 100 cells/μL before and after (‘primary comparison groups’) availability of AlereLAM. In pre-specified subgroups, we prospectively assessed AlereLAM-positive prevalence.

**Method:**

Clinicians prospectively performed AlereLAM in HIV-infected adults with TB symptoms and either CD4 ≤ 100 cells/μL or ‘seriously ill’ criteria. In a retrospective arm of equal duration, clinicians retrospectively collected data on HIV-infected adults with CD4 ≤ 100 cells/μL who initiated TB treatment.

**Results:**

A total of 115 prospectively eligible adults (of whom 55 had CD4 ≤ 100 cells/μL) and 77 retrospectively eligible patients were included. In the primary comparison groups, the retrospective and prospective arms had similar age and sex distribution. With availability of AlereLAM, the time to TB treatment decreased from a median of 4 to 3 days (p = 0.0557). With availability of AlereLAM, same-day TB treatment initiation rose from 9.1% to 32.7% (p = 0.0006). In those with CD4 ≤ 100 only, those with ‘seriously ill’ criteria only, and in those meeting either, or both, of these criteria, AlereLAM was positive in 10.5%, 21.9%, 34.8% and 48.4% respectively.

**Conclusion:**

Availability of AlereLAM led to more patients initiating same-day TB treatment. Using both CD4 ≤ 100 and ‘seriously ill’ criteria gave the greatest yield. Results of this study have informed local policy design.

## Introduction

In 2019, there were an estimated 10.0 million cases of tuberculosis (TB) worldwide.^1^ Those who had co-infection with HIV were at a disproportionately higher risk of death, despite TB being a curable disease. The World Health Organization (WHO) has highlighted that continued high rates of TB mortality relate to gaps in detection and diagnosis of this disease, as well as in poor linking of patients with care and treatment once TB is diagnosed. While sputum-based diagnostics have been the mainstay of TB diagnosis for decades, they have lower yield in people living with HIV (PLHIV) and are currently not able to provide a rapid answer at the bedside or in the clinic.

Lipoarabinomannan (LAM) is a component of the mycobacterial cell wall and has been assessed as a potential biomarker for active TB diagnosis in samples such as urine, sputum and serum – both with complex laboratory-based assays and in simple point-of-care devices.^2^ The Alere Determine TB LAM Ag assay (AlereLAM; Abbott, Chicago, IL, USA) is a lateral-flow rapid assay, which can provide a diagnosis within 30 min at the bedside on an easily collected urine sample. In unselected PLHIV in a recent Cochrane meta-analysis representing data from 3365 patients with 13% TB prevalence, AlereLAM had an estimated diagnostic sensitivity of 62% in patients who were hospitalised, and 31% in outpatients against a microbiological reference standard.^3^ In this same meta-analysis, AlereLAM had a specificity of 84% in inpatients compared to 95% in outpatients – also against a microbiological reference standard. While sputum-based diagnostics typically lose diagnostic yield in patients with lower CD4 counts, AlereLAM is consistently associated with greater sensitivity in this group, including in cohorts from similar settings as this study.^4^ Furthermore, in randomised, multi-country controlled trials at hospital level, availability of AlereLAM led to improved rates of survival in severely ill PLHIV.^5,6^

Despite the expanded antiretroviral access in South Africa, the proportion of patients presenting with advanced HIV disease (AHD; defined in adults as CD4 ≤ 200 cells/*µ*L or WHO stage 3/4 disease) has remained unchanged in recent years at 32% – 35%,^7^ including in 2019–2020 at approximately 34.6% (City of Cape Town data; personal communication). Furthermore, in resource-constrained settings, patients who qualify for hospital-level care where AlereLAM could be available might not always access this because of hospital bed shortages or difficulties in travelling to centralised care. Since 2019, the WHO has recommended that AlereLAM should be used to assist in the diagnosis of active TB in outpatient settings for PLHIV with CD4 ≤ 100 cells/*µ*L and in those with either ‘seriously ill’ criteria or signs and symptoms of TB.^8^ While sensitivity is low in outpatient settings, the WHO has motivated that making AlereLAM available to all qualifying PLHIV presenting for care at any level of the health system would be a step towards ensuring earlier TB diagnosis and reducing mortality in those at greatest risk.^1^

When new point-of-care tests are recommended and become available, the uptake and assessment of impact in real-world settings is not always straightforward.^9,10^ In early 2018, we began a pragmatic study with a before-after design to assess the impact of AlereLAM availability in the outpatient setting in three primary health care (PHC) clinics in Khayelitsha, Cape Town. We assessed whether giving clinicians access to AlereLAM could decrease the time to initiate TB treatment by allowing for greater same-day treatment initiation; we prospectively measured the prevalence of AlereLAM positivity in pre-specified subgroups and assessed how this introduction affected other diagnostic practices in a complex, demanding setting. This study was designed to assist with local AlereLAM policy development for the outpatient setting.

## Research methods and design

The study was initiated at three PHC clinics in two sub-districts of Cape Town, where there are high rates of HIV and TB. Because of staffing changes and an unfortunate fire that led to temporary closure of one of the clinics, the study could not be completed at one of the three initial clinics. We compared prospective and retrospective arms before and after AlereLAM was made available to clinicians. Eligibility criteria for this study were designed to reflect the WHO guidelines for patients qualifying for AlereLAM.^8^ In the prospective arm, eligible patients were consenting adults (≥18 years) with HIV, in whom a diagnosis of TB was suspected (based on WHO symptom screen) and who had either a CD4 ≤ 100 cells/*µ*L (within 6 months) and/or met criteria for being ‘seriously ill’ (as defined by any of respiratory rate > 30 breaths/min, heart rate > 120 beats/min, body mass index [BMI] ≤ 18.5 kg/m^2^, systolic blood pressure < 90 mmHg or being unable to walk unaided). In the retrospective arm, eligible patients were similarly adults (≥18 years) with HIV, who had a CD4 ≤ 100 cells/*µ*L (within 6 months) and were initiated on TB treatment. In both the retrospective and prospective arms, patients were excluded if they initiated TB treatment as inpatients, as this group would have already had access to AlereLAM in our setting. Of note, individual patients could only contribute to the data set once and a decision was made *a priori* to only include the first eligible presentation, even if an individual had presented on multiple occasions.

Before the start of the prospective arm, a training session was held where clinicians were taught how to perform and interpret the assay. The study had a pragmatic design and thereby did not systematically perform any additional TB testing, but training included the importance of performing additional tests irrespective of the AlereLAM result, for confirmation of *Mycobacterium tuberculosis* and for drug susceptibility testing. There are no on-site X-ray facilities at these clinics, but X-ray is accessible offsite approximately once on a weekly basis.

In the prospective arm, clinicians invited consent from eligible patients and recruitment continued until the minimum sample size was reached across all clinics. Retrospective data were collected through folder reviews of a period of equal duration to the prospective arm. Clinicians took 1 month to gain experience in performing the assay at their clinic, and to minimise the overlap of presenting periods between the retrospective and prospective arms (i.e. March 2018). The AlereLAM was performed by treating clinicians in their consulting rooms according to the manufacturer’s instructions. Briefly, 60 *µ*L of urine was applied to the sample pad and after 25 min test strips were read using the test’s reference scale card for grading with any band equal or greater in intensity than grade 1 deemed as positive.

Patients in both retrospective and prospective arms were followed up for 6 months. In both arms, all patients who had longer than 6 weeks between their presentation and TB treatment were excluded from the primary analysis as it would not be possible to delineate whether patients were presenting as part of the same or different clinical ‘episode’.

The primary objective was to compare the time to initiation of TB treatment between the retrospective and prospective arms in those patients who were initiated on treatment at PHC level and limited to those with CD4 ≤ 100 cells/*µ*L. The primary objective could only compare those meeting the CD4 criteria, in each arm, rather than those meeting ‘seriously ill’ criteria, in order to avoid introduction of misclassification bias with retrospective data collection. A secondary objective estimated the prevalence of AlereLAM positivity in the following groups in the prospective arm: those with CD4 ≤ 100 cells/*µ*L, those who met ‘seriously ill’ criteria and in those with either or both CD4 ≤ 100 cells/*µ*L and criteria for being ‘seriously ill’. The sample size calculation was done for the primary objective. We assumed that access to AlereLAM would decrease the mean time from presentation to treatment from 6 to 4 days. To ensure at least 80% statistical power with an alpha rate of 0.05, we required 63 participants per arm.

### Ethical considerations

This study was initiated after approval from the Faculty of Health Sciences Human Research Ethics Committee (HREC) (reference: #102/2018). Written informed consent was obtained from all the patients in the prospective arm. The HREC gave permission for clinicians to record data for the retrospective arm through folder reviews.

## Results

**FIGURE 1:**
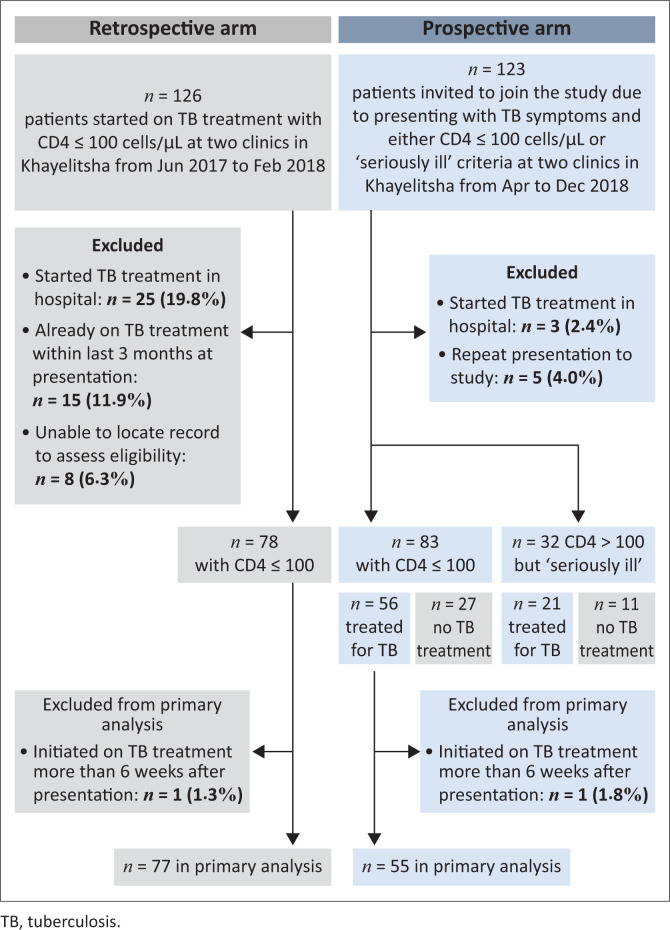
Flow diagram of patients.

In the 9-month period from June 2017 to February 2018, 126 patients received TB treatment and had a CD4 ≤ 100 cells/*µ*L at two PHC clinics in Khayelitsha (Figure 1). Of these, 25 (19.8%) were excluded from the study because of having initiated TB treatment in hospital, 15 (11.9%) had already received TB treatment in the 3 months prior to presentation and eight (6.3%) could not be included in the study because of medical records being misplaced – leaving 78 patients in the retrospective arm of this study. One of these 78 was started on TB treatment beyond the pre-specified 6-week cut-off period and therefore was not included in the primary analysis. A total of 61/77 (79.2%) had a known CD4 count on the day that TB treatment was initiated.

Between April and December 2018, 123 eligible patients with TB symptoms gave written consent to take part in the study (Figure 1). Within the prospective arm, 56 had CD4 ≤ 100 cells/*µ*L and were treated for TB and 27 had CD4 ≤ 100 cells/*µ*L but were not started on TB treatment. One of these 56 was started on TB treatment beyond the pre-specified 6-week cut-off period and therefore was not included in the primary analysis. A further 32 in the prospective arm had CD4 > 100 but met the criteria for being ‘seriously ill’. A total of 36/55 (65.5%) patients in the prospective arm had a known CD4 count on the day that AlereLAM was performed.

The 77 patients in the retrospective arm were compared with the 55 patients in the prospective arm with CD4 counts ≤ 100 cells/*µ*L, for the primary objective of this study. They had a similar age distribution (retrospective: median 36 years; prospective: median 35 years) and sex distribution (retrospective: 46.8% female; prospective: 50.9% female) (Table 1). There were more new HIV diagnoses in the retrospective (32.5%) group than in the prospective (10.9%) group. The retrospective arm had more PLHIV who were antiretroviral therapy (ART)-naïve (46.8%) compared to the prospective arm (16.4%). There were more patients meeting the criteria for being ‘seriously ill’ in the prospective (87.3%) group than in the retrospective (50.6%) group.

**TABLE 1:** Baseline characteristics of the study population.

Characteristic	Prospective arm (all) *n* = 115				Prospective arm (included in primary analysis) *n* = 55				Retrospective arm *n* = 77				p*
	Median	IQR	*n*	%	Median	IQR	*n*	%	Median	IQR	*n*	%	
**Demographics**													
Age (years)	35	31–43	-	-	35	31–41	-	-	36	30–42	-	-	0.4797
Females	-	-	59	51.3	-	-	28	50.9	-	-	36	46.8	0.6376
**HIV status**													
Newly diagnosed HIV	-	-	15	13.0	-	-	6	10.9	-	-	25	32.5	0.0040
Known HIV diagnosis	-	-	100	87.0	-	-	49	89.1	-	-	52	67.5	-
**ART status**													
ART-naïve	-	-	20	17.4	-	-	9	16.4	-	-	36	46.8	0.0003
Currently on ART	-	-	36	31.3	-	-	16	29.1	-	-	8	10.4	-
ART interrupted	-	-	59	51.3	-	-	30	54.5	-	-	33	42.9	-
**Other clinical descriptors**													
CD4 count (cells/*μ*L)	61	28–117	-	-	38	20–65	-	-	47	30–73	-	-	0.1776
Known history of TB treatment	-	-	57	49.6	-	-	25	45.5	-	-	30	39.0	0.4556
Met ‘seriously ill’ criteria†	-	-	96	83.5	-	-	48	87.3	-	-	39	50.6	< 0.0001

ART, antiretroviral therapy; IQR, interquartile range; TB, tuberculosis.

* , *p*-values represent comparison between patients in prospective arm included in primary analysis and retrospective arm.

† , ‘Seriously ill’ criteria included respiratory rate > 30 breaths/min, heart rate > 120 beats/min, BMI ≤ 18.5 kg/m^2^, systolic blood pressure < 90 mmHg or being unable to walk unaided.

**FIGURE 2:**
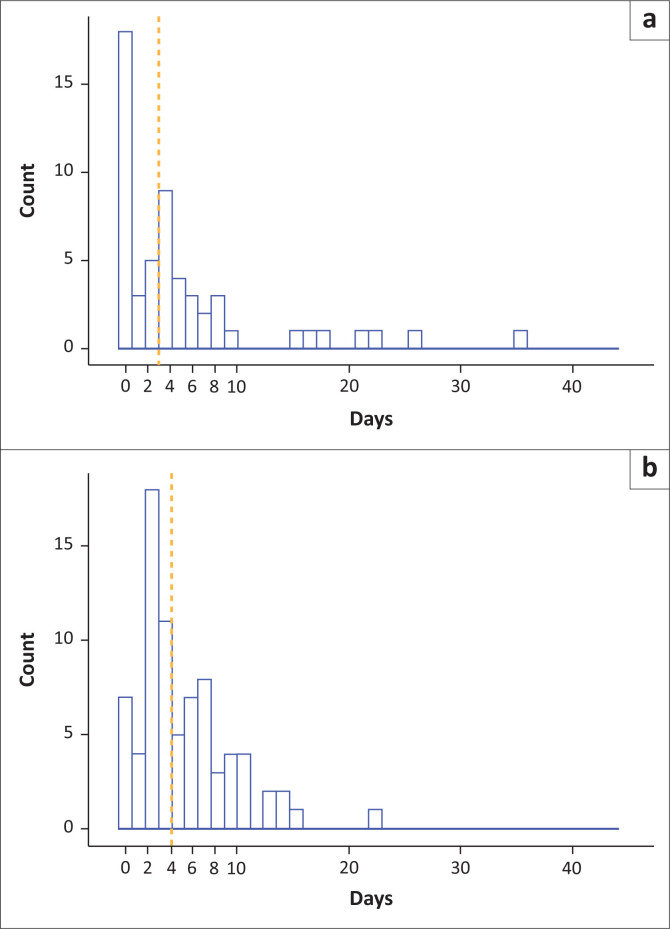
Histogram of the number of patients initiating TB treatment per day, over days 0–42 in the study, in (a) the prospective arm meeting criteria for inclusion in primary analysis and (b) the retrospective arm. Orange dashed line represents the median.

Once AlereLAM had become available (i.e. prospective arm), there was a median of 1 day (interquartile range [IQ]): 0–5 days) between presentation and clinicians performing the AlereLAM. Before AlereLAM availability, patients were initiated on TB treatment at a median of 4 days (IQR: 2–7 days) after presentation compared to a median of 3 days once AlereLAM had become available (IQR: 0–6 days) (*p* = 0.0557) (Figure 2).

A total of 7/77 (9.1%) and 18/55 (32.7%) of patients were initiated on TB treatment on the same day as presentation before and after AlereLAM availability, respectively (*p* = 0.0006).

Before and after the introduction of AlereLAM, patients had a similar number of microbiological tests for *M. tuberculosis* (*p* = 0.8535) and a similar proportion had chest X-rays performed (*p* = 0.5143) as part of their work-up for TB (Table 2). At the time of TB treatment initiation, 54.5% of patients in the retrospective arm were initiated on this treatment based on clinical and/or radiological features only and this empiric treatment declined to 30.9% in the prospective arm (*p* = 0.0071). Before AlereLAM availability, 85.7% (66/77) of patients had a rapid test performed on sputum (i.e. GeneXpert or Smear) compared to 66.1% (76/115) after AlereLAM became available. Non-AlereLAM urine TB tests became more common after AlereLAM became available, with 14.3% (11/77) having urine sent for mycobacterial culture before AlereLAM availability compared to 32.2% (23/115) after AlereLAM availability. Of note, GeneXpert testing on urine is not currently routinely available in this setting.

**TABLE 2:** Tuberculosis diagnostic tests done before and after the introduction of AlereLAM in the primary comparison groups.

Diagnostic characteristic	Prospective arm *n* = 55				Retrospective arm *n* = 77			
	Median	IQR	*n*	%	Median	IQR	*n*	%
**Evidence towards TB treatment at day of initiation†**								
Radiological	-	-	17	30.9	-	-	42	54.5
GeneXpert	-	-	3	5.5	-	-	26	33.8
AlereLAM	-	-	40	60.0	-	-	-	-
Mycobacterial culture	-	-	1	1.8	-	-	1	1.3
Smear	-	-	0	0.0	-	-	4	5.2
Other investigation, for example, pleural fluid ADA	-	-	1	1.8	-	-	7	9.1
Based on radiological and/or clinical features only	-	-	17	30.9	-	-	42	54.5
**Microbiological investigations done per patient‡**								
Total tests	2	2–4	-	-	2	2–3	-	-
Total positive tests	0	0–1	-	-	1	0–2	-	-
Had ≥ 1 positive GeneXpert/Smear/TB culture	-	-	24	43.6	-	-	53	68.8
Had sputum rapid test§	-	-	39	70.9	-	-	66	85.7
Had sputum TB culture	-	-	25	45.5	-	-	35	45.5
Had urine smear	-	-	13	23.6	-	-	1	1.3
Had urine TB culture	-	-	19	34.5	-	-	11	14.3
Had non-sputum and non-urine rapid test	-	-	6	10.9	-	-	11	14.3
Had non-sputum and non-urine TB culture	-	-	11	20.0	-	-	16	20.8
Had chest X-ray	-	-	29	52.7	-	-	45	58.4
**Time (days) to diagnostic test result**								
Presentation to earliest positive microbiology result	14	3–20	-	-	6	1–15	-	-
Presentation to AlereLAM	1	0–5	-	-	-	-	-	-

ADA, adenosine deaminase; IQR, interquartile range; TB, tuberculosis.

† , Note that patients can contribute to multiple groups, unless specified;

‡ , Not including AlereLAM;

§ , A rapid test refers to either GeneXpert or smear.

**FIGURE 3:**
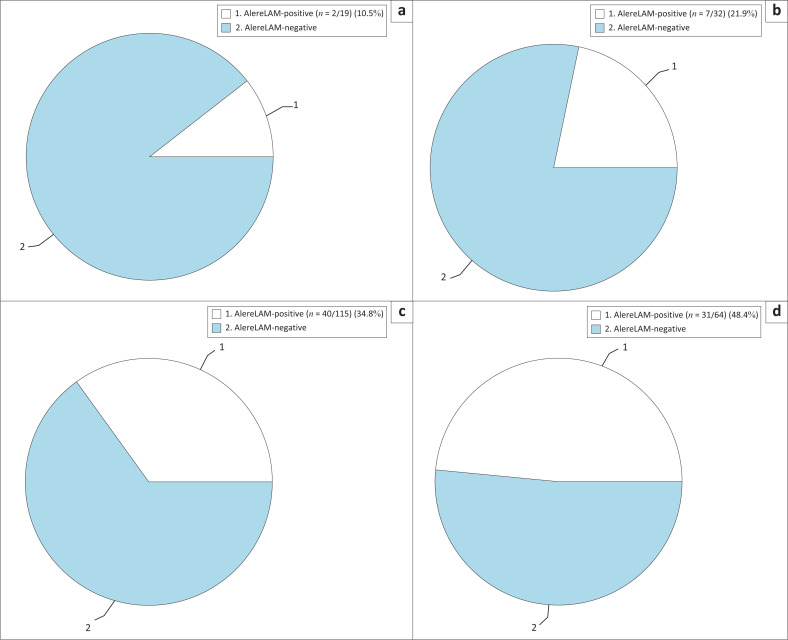
AlereLAM-positive prevalence in (a) patients with CD4 ≤ 100 but not meeting ‘seriously ill’ criteria, (b) those with ‘seriously ill’ criteria but CD4 > 100, (c) those with either CD4 ≤ 100 or ‘seriously ill’ criteria, and (d) those meeting both CD4 ≤ 100 and ‘seriously ill’ criteria.

In the prospective arm, irrespective of eventual TB treatment, the prevalence of AlereLAM positivity in those with CD4 ≤ 100 cells/*µ*L and not meeting ‘seriously ill’ criteria was 10.5% (*n* = 2/19) (Figure 3). In those who met the ‘seriously ill’ criteria but had CD4 > 100 cells/*µ*L, the prevalence of AlereLAM positivity was 21.9% (*n* = 7/32) and in this group the median CD4 was 148 cells/*µ*L, with a range from 103 cells/*µ*L to 469 cells/*µ*L. By including those who met either CD4 ≤ 100 cells/*µ*L or ‘seriously ill’ criteria, this yielded an AlereLAM positive prevalence of 34.8% (*n* = 40/115). The highest yield for AlereLAM positive prevalence was in those PLHIV meeting both CD4 ≤ 100 cells/*µ*L and ‘seriously ill’ criteria at 48.4% (*n* = 31/64).

In both the retrospective and prospective arms, patients were not eligible for the study if their TB treatment initiation was at hospital level. Within the 6-month follow-up period, risk of hospitalisation that did not include TB treatment initiation was similar in the retrospective (22/78; 28.2%) and prospective (38/115; 33.0%) arms (*p* = 0.4761). Four patients (4/78; 5.1%) in the retrospective arm and eight patients (8/115; 7.0%) in the prospective arm died during the 6-month follow-up period (*p* = 0.7649). In the prospective arm where AlereLAM was available, 3/40 (7.5%) of AlereLAM-positive patients died and 5/75 (6.7%) of AlereLAM-negative patients died.

## Discussion

In a high-burden TB and HIV setting, the availability of AlereLAM at ambulatory level meant that more patients with both HIV and TB who were presenting for care with low CD4 counts were initiated on TB treatment on the same day as presentation, and the proportion initiating TB treatment empirically was nearly halved. Importantly, the study found that the greatest yield in detecting AlereLAM-positive patients was when the combination of CD4 and ‘seriously ill’ criteria was used, emphasising the importance of implementing the full set of criteria for AlereLAM, especially when CD4 counts might not be known on the day of presentation.

While the findings of this study have been used to inform local policy design, there are a number of key limitations to consider. Firstly, this study was conducted in clinics in Khayelitsha, Cape Town and the results might not be generalisable to other areas that have different burdens of TB and HIV. Secondly, there could have been patients in the prospective arm of this study who were eligible for AlereLAM but were missed because of oversight or having already been initiated on TB treatment empirically before they were known to be eligible for AlereLAM. However, this would have likely meant that we underestimated the effect of AlereLAM on decreasing time to treatment initiation in comparison to the retrospective arm, rather than overestimated it. Furthermore, our sample size was too small to make robust conclusions regarding the exploratory analyses of associations with the clinical outcomes of hospitalisation and death. While a before-after study design comes with inherent limitations in comparison to the gold standard of a randomised controlled trial, the former allowed for policy-informing evidence to be accurately generated within a short time and with limited resources.

Other studies have been performed to assess the impact of AlereLAM on time to initiation of TB treatment in an outpatient setting.^11,12,13^ The multi-centre TB-NEAT trial performed AlereLAM on biobanked urine samples and concluded that AlereLAM results would have no impact on decreasing time to initiation of TB treatment if chest X-ray facilities are available on-site and where rates of empiric TB treatment are high.^13^ Despite a high burden of TB in our study’s community and generally higher healthcare resources in South Africa than some other comparable high-burden settings, the clinics in this study do not have access to on-site chest X-ray. With availability of AlereLAM, we saw that the rates of empiric TB treatment decreased significantly, and the number of patients being initiated on TB treatment on the same day as presentation tripled. Based on cohorts recruited in South Africa and other African countries, modelling studies have estimated that implementation of AlereLAM within TB diagnostic algorithms is also cost-effective.^14,15^

Simplifying the criteria for AlereLAM as far as possible would likely improve its uptake and implementation, but this needs to be balanced against diagnostic yield of the criteria considered, which in turn impacts cost-effectiveness. In comparison to our study, others have reported on AlereLAM-positive prevalence in ambulatory PLHIV irrespective of CD4 or ‘seriously ill’ criteria and estimated this prevalence to be much lower (from 13.0%^16^ to 16.9%^11^). Our higher AlereLAM-positive prevalence (34.8%) is likely because of selecting patients who had either CD4 ≤ 100 cells/*µ*L or criteria for being ‘seriously ill’. While CD4 criteria are informed by the greater diagnostic sensitivity in this group, CD4 counts might not always be available on the day of presentation for care. However, the criteria for being ‘seriously ill’ rely on vital signs that can be performed in any setting and our study showed that implementing the combination of these criteria had the greatest yield.

The effects of introducing a point-of-care diagnostic test into a new complex setting and diagnostic environment are important to consider. With the limited sensitivity of AlereLAM and its inability to diagnose drug resistance, further diagnostic work-up is critical, with the current recommendation being that AlereLAM should be performed in conjunction with a sputum GeneXpert.^8^ Once AlereLAM became available to clinicians, they performed a similar number of microbiological tests and chest X-rays overall but tended to perform fewer sputum rapid tests (i.e. GeneXpert or smear) and a similar proportion of sputum mycobacterial cultures, but double the number of mycobacterial cultures on urine. Noting that urine is an easier sample to collect and clinicians had already collected this sample for the AlereLAM, this might have been the reason as to why they performed more mycobacterial cultures on urine. However, the yield of mycobacterial culture of urine for pulmonary or extra-pulmonary TB diagnosis is very low (< 10%).^17,18^ Furthermore, it is preferable to perform a GeneXpert test and not a mycobacterial culture alone as it takes several weeks before a culture result becomes available and this could lead to significant delays in TB or rifampicin resistance diagnosis. This pragmatic study’s findings highlight the importance of clear guidelines regarding further diagnostic work-up, whether a patient’s AlereLAM result is positive or negative.

In line with the WHO’s guidance to make AlereLAM accessible as a means to decrease TB mortality in PLHIV, AlereLAM should be made available to all patients meeting criteria for testing in outpatient settings. This pragmatic study highlights the potential benefits and consequences when rolling out this point-of-care test in a new setting that should be considered by both policymakers and clinicians.

## Conclusion

With the availability of AlereLAM, the percentage of patients being initiated on TB treatment on the same day as presentation rose from 9.1% to 32.7% and the median time for initiation of TB treatment was 3 days compared to 4 days. Using CD4 ≤ 100 cells/*μ*L and ‘seriously ill’ testing criteria gave the highest yield of AlereLAM-positive patients. These findings highlight the utility of this rapid point-of-care TB test at PHC clinics for ill patients with HIV who are at high risk of mortality from TB.
